# Nonlocal Vibration Analysis of a Nonuniform Carbon Nanotube with Elastic Constraints and an Attached Mass

**DOI:** 10.3390/ma14133445

**Published:** 2021-06-22

**Authors:** Maria Anna De Rosa, Maria Lippiello, Enrico Babilio, Carla Ceraldi

**Affiliations:** 1 School of Engineering, University of Basilicata, Viale dell’Ateneo Lucano 10, 85100 Potenza, Italy; 2 Department of Structures for Engineering and Architecture, University of Naples “Federico II”, Via Forno Vecchio 36, 80134 Naples, Italy; maria.lippiello@unina.it (M.L.); enrico.babilio@unina.it (E.B.); ceraldi@unina.it (C.C.)

**Keywords:** nanosensor, nonlocal elasticity, frequency analysis, elastically restrained beams, DQM

## Abstract

Here, we consider the free vibration of a tapered beam modeling nonuniform single-walled carbon nanotubes, i.e., nanocones. The beam is clamped at one end and elastically restrained at the other, where a concentrated mass is also located. The equation of motion and relevant boundary conditions are written considering nonlocal effects. To compute the natural frequencies, the differential quadrature method (DQM) is applied. The influence of the small-scale parameter, taper ratio coefficient, and added mass on the first natural frequency is investigated and discussed. Some numerical examples are provided to verify the accuracy and validity of the proposed method, and numerical results are compared to those obtained from exact solution. Since the numerical results are in excellent agreement with the exact solution, we argue that DQM provides a simple and powerful tool that can also be used for the free vibration analysis of carbon nanocones with general boundary conditions for which closed-form solutions are not available in the literature.

## 1. Introduction

Carbon-based nanostructures have been intensively researched due to their outstanding properties. Among others, carbon nanotubes (CNTs) and nanocones (CNCs), since their discovery dating back to 1991 [1] and 1994 [2], respectively, have inspired many studies to understand their electromechanical [3], mechanical, and thermal properties [4]; to analyze vibrations of fluid flow in single-walled CNTs [5]; and exploit their potential in applications in nanoelectronics [6] or as gas sensors [7], mass sensors [8], nanomechanical sensors [9], or in the preparation of hierarchical materials by chemical grafting of CNTs onto carbon fibers [10], to cite but a few.

The many different approaches already available to study the behavior of nanostructures can be grouped in two classes: one at the atomistic level, the other at the continuum level. The latter have attracted huge attention, as those in the former class require often difficult and time-consuming computations [11], although atomistic tools may appear to be the most suitable for nanosized structures. Among continuum approaches, beam models have been demonstrated to be cost-effective. However, classical beam theories, as Euler–Bernoulli or Timoshenko beams, or even higher-order theories [12], may be inadequate because they do not capture the influence of size effects. To overcome this drawback, models incorporating nonlocal effects are often considered, such as those based on the nonlocal elasticity theory developed by Eringen (see, in particular, [13,14]).

However, recent investigations have led to the conclusion that the elastic problems based on the Eringen strain-driven model are ill-posed [15]. For bounded structural domains, constitutive boundary conditions must be added to recover equivalence with nonlocal strain-driven integral law. The differential elastic law leads to a well-posed structural problem whose solution may paradoxically reproduce the local elastic solution, see, e.g., [16,17,18]. The well-posedeness of elastic problems based on a nonlocal integral model can be recovered by adopting a stress-driven formulation [19,20,21].

The use of nonlocal continuum theory in the field of nanotechnology was first reported in [22] and further applications have been employed in analyzing the buckling and vibration problems in CNTs, by applying Euler–Bernoulli and Timoshenko beam theories [23,24,25,26]. Wave propagation in CNTs was studied with nonlocal elastic Euler–Bernoulli and Timoshenko beam models in [27]. The constitutive relations of nonlocal elasticity theory for the analysis of CNTs modelled as Euler–Bernoulli beams, Timoshenko beams, or as cylindrical shells are presented in [28]. The scale effect on static deformation of micro- and nano-rods or tubes was studied by [29] through nonlocal Euler–Bernoulli and Timoshenko beam theories, with the results showing that the scale effect, which would not manifest itself for micro-structures with a length in the order of micrometers, would be noticeable in the static response of nano-structures. Still based on the nonlocal Euler–Bernoulli beam theory, the effects of taper ratio coefficient, small-scale parameter, and viscoelastic behavior on the resonant frequencies of CNCs was discussed in [30]. Employing the differential quadrature method (DQM), the vibration response of nanocantilever was studied [31] and a nonlocal-elasticity-based formulation for the axial vibration analysis of tapered nanorods was constructed [32].

Dealing with free vibration analysis of a circular hollow nanobeam, clamped at one end and elastically restrained at the other, that models a mass-sensor composed of a CNT or a CNC, depending on the considered taper ratio, a nanobeam loaded by a lumped mass is considered in this paper. In addition to nano-sized mass-sensors, the topic of vibrations of lumped-mass-loaded structures is relevant in different engineering-related fields, such as acoustics [33,34].

This paper is organized as follows. Details about the equation of motion of tapered nanobeams and relevant boundary conditions written considering nonlocal effects are provided in Section 2. Next, Section 3 describes the differential quadrature method (DQM) that is adopted in this paper to compute the first natural frequency of the analyzed nanobeams. The influence of the small-scale parameter, taper ratio coefficient, and added mass on the first, natural, dimensionless frequency is investigated in Section 4 to assess the accuracy and validity of the proposed method. The results complement those previously reported in [35], where the convergence of the method was validated through known exact solutions. With low computational effort, problems characterized by boundary conditions and geometries for which closed-form solutions are currently not available may be considered. Some concluding remarks are provided in Section 5.

## 2. Formulation of the Problem

**Figure 1:**
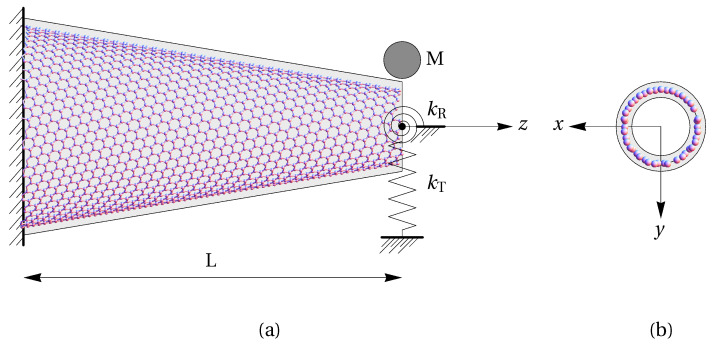
Geometry of a nanocone having an apex angle of $19.18^{\circ }$; length of 80 Å; and radii of 21.8 Å and 8 Å at the anchored side and at the tip, respectively, superimposed on the corresponding tapered beam model (**a**) and front view of the tip of the nanocone superimposed on the hollow circular cross-section of the beam model (**b**). The wall thickness of the cross-section at the continuum level is accepted as 3.4 Å.

Let us consider the carbon nanocone (CNC) sensor shown in Figure 1. The CNC, which is a nonuniform or tapered carbon nanotube (CNT), is anchored to a fixed support and interacts at the tip with the surrounding environment and a molecule. The CNC (with an apex angle of $19.18^{\circ }$ in Figure 1) is modeled, at a continuum level, as a tapered beam having a hollow, circular cross-section. The anchorage is modeled by a clamp and the tip interactions by an axial spring of stiffness $k_{T}$ and an angular torsion spring of stiffness $k_{R}.$ The molecule is considered as a lumped mass *M*. The length *L* of the beam coincides with the length of the CNC (80 Å in Figure 1) and the radii of the end cross-sections are equal to the corresponding average radii of the CNC (21.8 Å and 8 Å in Figure 1).

In this paper, the wall thickness of the cross-section is assumed to be 3.4 Å [36], which is equal to the separation between the walls of multi-walled CNTs [37]. However, other sizes have been considered previously, such as 1.32 Å, the length of the π orbital [38]; or 1.54 Å, the covalent diameter of the carbon atom [39].

In agreement with Figure 1, the origin of the reference frame is set coincident with the centroid of the clamped cross-section, whose plane contains the axes *x* and *y* while the coordinate *z* is along the beam centerline. By denoting the time variable as *t* and following [40], where the free vibrations of a CNT were analyzed, the governing equation of motion for a nonuniform nanobeam and the corresponding boundary conditions can be written by using the Hamilton’s variational principle as

(1) $$\begin{array}{c} \frac{\partial^{2}}{\partial z^{2}}\left(EI\left(z\right)\frac{\partial^{2}v\left(z,t\right)}{\partial z^{2}}-{\left(e_{0}a\right)}^{2}\rho A\left(z\right)\frac{\partial^{2}v\left(z,t\right)}{\partial t^{2}}\right)+\rho A\left(z\right)\frac{\partial^{2}v\left(z,t\right)}{\partial t^{2}}=0\;, \end{array}$$

(2) $$\begin{array}{c} v(0,t)=0\;, \end{array}$$

(3) $$\begin{array}{c} {\left.\frac{\partial v(z,t)}{\partial z}\right|}_{z=0}=0\;, \end{array}$$

(4) $$\begin{array}{c} EI\left(L\right){\left.\frac{\partial^{2}v\left(z,t\right)}{\partial z^{2}}\right|}_{z=L}-{\left(e_{0}a\right)}^{2}\rho A\left(L\right){\left.\frac{\partial^{2}v\left(z,t\right)}{\partial t^{2}}\right|}_{z=L}+k_{R}{\left.\frac{\partial v(z,t)}{\partial z}\right|}_{z=L}=0\;, \end{array}$$

(5) $$\begin{array}{c} {\left.\frac{\partial }{\partial z}\left(EI\left(z\right)\frac{\partial^{2}v\left(z,t\right)}{\partial z^{2}}-{\left(e_{0}a\right)}^{2}\rho A\left(z\right)\frac{\partial^{2}v\left(z,t\right)}{\partial t^{2}}\right)\right|}_{z=L}-M{\left.\frac{\partial^{2}v\left(z,t\right)}{\partial t^{2}}\right|}_{z=L}-k_{T}v\left(L,t\right)=0\;, \end{array}$$

where v(z,t) is the transverse displacement, ρ is the mass density, *E* is Young’s modulus, A(z) is the cross-sectional area, I(z) is the second moment of area, $e_{0}$ is a constant depending on the material, and *a* is an internal characteristic length, such as the inter-atomic distance, which is 1.42 Å in case of carbon–carbon bonds [41].

Assuming that

(6) v(z,t)=v(z)cosωt

holds, with ω being the natural frequency of vibrations, Equations (1)–(5) can be rewritten as

(7) $$\begin{array}{c} E\frac{\partial^{2}}{\partial z^{2}}\left(I\left(z\right)\frac{\partial^{2}v\left(z\right)}{\partial z^{2}}\right)+\omega^{2}{\left(e_{0}a\right)}^{2}\rho \frac{\partial^{2}\left(A(z)v(z)\right)}{\partial z^{2}}-\omega^{2}\rho A\left(z\right)v\left(z\right)=0\;, \end{array}$$

(8) $$\begin{array}{c} v(0)=0\;, \end{array}$$

(9) $$\begin{array}{c} {\left.\frac{\partial v(z)}{\partial z}\right|}_{z=0}=0\;, \end{array}$$

(10) $$\begin{array}{c} EI\left(L\right){\left.\frac{\partial^{2}v\left(z\right)}{\partial z^{2}}\right|}_{z=L}+\omega^{2}{\left(e_{0}a\right)}^{2}\rho A\left(L\right)v\left(L\right)+k_{R}{\left.\frac{\partial v(z)}{\partial z}\right|}_{z=0}=0\;, \end{array}$$

(11) $$\begin{array}{c} E{\left.\frac{\partial }{\partial z}\left(I\left(z\right)\frac{\partial^{2}v\left(z\right)}{\partial z^{2}}\right)\right|}_{z=L}+\omega^{2}{\left(e_{0}a\right)}^{2}\rho {\left.\frac{\partial \left(A(z)v(z)\right)}{\partial z}\right|}_{z=L}+\left(\omega^{2}M-k_{T}\right)v\left(L\right)=0\;. \end{array}$$

On introducing the dimensionless taper-ratio coefficient ϵ and the function

(12) $$g\left(z\right)=1+\epsilon \frac{z}{L}\;,$$

the cross-sectional area and second moment of area are assumed to satisfy

(13) $$\begin{array}{c} A\left(z\right)=A_{0}\;g{\left(z\right)}^{q_{2}}\;, \end{array}$$

(14) $$\begin{array}{c} I\left(z\right)=I_{0}\;g{\left(z\right)}^{q_{1}+2}\;, \end{array}$$

where $q_{1}$ and $q_{2}$ are shape factors and $A_{0}=A\left(0\right)$ and $I_{0}=I\left(0\right)$ are set.

Note that ϵ must be greater than −1 to prevent the beam profile from tapering to zero as it passes from one end to the other; ϵ=0 corresponds to the uniform profile and ϵ>0 yields an increasing profile.

Substituting Equations (13) and (14) into Equation (7), we obtain

(15) $$\begin{array}{cc} \; & EI_{0}\left(\frac{\epsilon^{2}}{L^{2}}\left(q_{1}+1\right)\left(q_{1}+2\right)g{\left(z\right)}^{q_{1}}\frac{\partial^{2}v\left(z\right)}{\partial z^{2}}+2\frac{\epsilon }{L}\left(q_{1}+2\right)g{\left(z\right)}^{q_{1}+1}\frac{\partial^{3}v\left(z\right)}{\partial z^{3}}+g{\left(z\right)}^{q_{1}+2}\frac{\partial^{4}v\left(z\right)}{\partial z^{4}}\right)\\ + & \omega^{2}{\left(e_{0}a\right)}^{2}\rho A_{0}\left(\frac{\epsilon^{2}}{L^{2}}q_{2}\left(q_{2}-1\right)g{\left(z\right)}^{q_{2}-2}v\left(z\right)+2\frac{\epsilon }{L}q_{2}g{\left(z\right)}^{q_{2}-1}\frac{\partial v(z)}{\partial z}+g{\left(z\right)}^{q_{2}}\frac{\partial^{2}v\left(z\right)}{\partial z^{2}}\right)\\ - & \omega^{2}\rho A_{0}\;g{\left(z\right)}^{q_{2}}v\left(z\right)=0\;. \end{array}$$

The boundary conditions (8)–(11) are rewritten accordingly. In particular, Equations (10) and (11) take the form

(16) $$\begin{array}{c} EI_{0}{\left(1+\epsilon \right)}^{q_{1}+2}{\left.\frac{\partial^{2}v\left(z\right)}{\partial z^{2}}\right|}_{z=L}+\omega^{2}{\left(e_{0}a\right)}^{2}\rho A_{0}{\left(1+\epsilon \right)}^{q_{2}}v\left(L\right)+k_{R}{\left.\frac{\partial v(z)}{\partial z}\right|}_{z=L}=0\;, \end{array}$$

(17) $$\begin{array}{c} \begin{array}{cc} & EI_{0}\left(\frac{\epsilon }{L}\left(2+q_{1}\right){\left(1+\epsilon \right)}^{q_{1}+1}{\left.\frac{\partial^{2}v\left(z\right)}{\partial z^{2}}\right|}_{z=L}+{\left(1+\epsilon \right)}^{q_{1}+2}{\left.\frac{\partial^{3}v\left(z\right)}{\partial z^{3}}\right|}_{z=L}\right)\\ + & \omega^{2}{\left(e_{0}a\right)}^{2}\rho A_{0}\left(\frac{\epsilon }{L}\;q_{2}{\left(1+\epsilon \right)}^{q_{2}-1}v\left(L\right)+{\left(1+\epsilon \right)}^{q_{2}}{\left.\frac{\partial v(z)}{\partial z}\right|}_{z=L}\right)\\ + & \left(\omega^{2}M-k_{T}\right)v\left(L\right)=0\;. \end{array} \end{array}$$

## 3. Solution by the Differential Quadrature Method

The solution of Equation (15) is numerically approximated using the DQM [42,43,44,45].

By virtue of the remapping rules

(18) $$\begin{array}{c} \zeta =2\frac{z}{L}-1\;, \end{array}$$

(19) $$\begin{array}{c} f\left(\zeta \right)=1+\frac{\epsilon }{2}\left(\zeta +1\right)\;, \end{array}$$

with ζ∈[−1,1] being the dimensionless counterpart of z∈[0,L], Equation (15) is rewritten as

(20) $$\begin{array}{cc} \; & 4\epsilon^{2}\left(q_{1}+1\right)\left(q_{1}+2\right)f{\left(\zeta \right)}^{q_{1}}\frac{\partial^{2}v\left(\zeta \right)}{\partial \zeta^{2}}+16\epsilon \left(q_{1}+2\right)f{\left(\zeta \right)}^{q_{1}+1}\frac{\partial^{3}v\left(\zeta \right)}{\partial \zeta^{3}}+16f{\left(\zeta \right)}^{q_{1}+2}\frac{\partial^{4}v\left(\zeta \right)}{\partial \zeta^{4}}\\ = & \Omega^{2}f{\left(\zeta \right)}^{q_{2}}v\left(\zeta \right)-\eta^{2}\Omega^{2}\left(\epsilon^{2}q_{2}\left(q_{2}-1\right)f{\left(\zeta \right)}^{q_{2}-2}v\left(\zeta \right)-4\epsilon q_{2}f{\left(\zeta \right)}^{q_{2}-1}\frac{\partial v(\zeta )}{\partial \zeta }-4f{\left(\zeta \right)}^{q_{2}}\frac{\partial^{2}v\left(\zeta \right)}{\partial \zeta^{2}}\right)\;, \end{array}$$

where the dimensionless quantities

(21) $$\eta =\frac{e_{0}a}{L}\;,\;\Omega =\omega L^{2}\sqrt{\frac{\rho \;A_{0}}{E\;I_{0}}}\;,\;\lambda =\frac{M}{\rho A_{0}L}\;$$

are set. In particular, with reference to the parameter λ, notice that λ=0 indicates the absence of external molecules, whereas λ=1 denotes that the molecule has the same mass as the nanobeam.

Then, the boundary conditions, Equations (8), (9), (16), and (17), become

(22) $$\begin{array}{c} v(-1)=0\;, \end{array}$$

(23) $$\begin{array}{c} {\left.\frac{\partial^{2}v\left(\zeta \right)}{\partial \zeta^{2}}\right|}_{\zeta =-1}=0\;, \end{array}$$

(24) $$\begin{array}{c} {\left(1+\epsilon \right)}^{q_{1}+2}{\left.\frac{\partial^{2}v\left(\zeta \right)}{\partial \zeta^{2}}\right|}_{\zeta =1}+K_{R}{\left.\frac{\partial v(\zeta )}{\partial \zeta }\right|}_{\zeta =1}=-\Omega^{2}\frac{\eta^{2}}{4}{\left(1+\epsilon \right)}^{q_{2}}v\left(1\right)\;, \end{array}$$

(25) $$\begin{array}{c} \begin{array}{cc} & \frac{\epsilon }{2}\left(2+q_{1}\right){\left(1+\epsilon \right)}^{q_{1}+1}{\left.\frac{\partial^{2}v\left(\zeta \right)}{\partial \zeta^{2}}\right|}_{\zeta =1}+{\left(1+\epsilon \right)}^{q_{1}+2}{\left.\frac{\partial^{3}v\left(\zeta \right)}{\partial \zeta^{3}}\right|}_{\zeta =1}-K_{T}v\left(1\right)\\ = & -\Omega^{2}\eta^{2}\left(\epsilon \frac{1}{8}q_{2}{\left(1+\epsilon \right)}^{q_{2}-1}v\left(1\right)+\frac{1}{4}{\left(1+\epsilon \right)}^{q_{2}}{\left.\frac{\partial v(\zeta )}{\partial \zeta }\right|}_{\zeta =1}\right)-\Omega^{2}\frac{\lambda }{8}v\left(1\right)\;, \end{array} \end{array}$$

with

(26) $$K_{T}=\frac{k_{T}L}{8EI_{0}}\;,\;K_{R}=\frac{k_{R}L^{3}}{2EI_{0}}\;.$$

To discretize Equation (20), the interval [−1,1] is divided into *n* segments defined using *n* + 1 points located at

(27) $$\zeta_{i}=\frac{2(i-1)-n}{n}\;,\;i=1,2,\ldots ,n+1\;,$$

and the set of n+7 nodal unknowns, namely, the displacement at each nodal point and the first three derivatives at the end points, are stored in the vector

(28) $$w^{T}=\left\{v_{1},v_{1}^{\prime },v_{1}^{\prime \prime },v_{1}^{\prime \prime \prime },v_{2},v_{3},\ldots ,v_{n-1},v_{n},v_{n+1},v_{n+1}^{\prime },v_{n+1}^{\prime \prime },v_{n+1}^{\prime \prime \prime }\right\}\;,$$

where $v_{i}$ and the prime symbol ${(}^{\prime })$ are $v(\zeta_{i})$ and the derivative with respect to ζ, respectively.

The displacement v(ζ) is approximated as

(29) $$v\left(\zeta \right)=\alpha \left(\zeta \right)C=\sum_{i=1}^{n+7}\alpha_{i}C_{i}\;,$$

where α(ζ) is a row vector of monomials as

(30) $$\alpha \left(\zeta \right)=\left(1,\zeta ,\zeta^{2},\ldots ,\zeta^{n+6}\right)\;,$$

and C is a column vector of Lagrangian coordinates. The derivatives of Equation (29) are

(31) $$v^{\prime }\left(\zeta \right)=\alpha^{\prime }\left(\zeta \right)C\;,\;v^{\prime \prime }\left(\zeta \right)=\alpha^{\prime \prime }\left(\zeta \right)C\;,\;v^{\prime \prime \prime }\left(\zeta \right)=\alpha^{\prime \prime \prime }\left(\zeta \right)C\;.$$

Evaluating Equations (29) and (31) at the nodal coordinates given by Equation (27) and substituting into Equation (28), we obtain

(32) $$w=N_{0}C\;,$$

where $N_{0}$ is a (n+7)×(n+7) matrix whose rows are described by vectors

(33) $$\alpha \left(\zeta_{1}\right),\alpha^{\prime }\left(\zeta_{1}\right),\alpha^{\prime \prime }\left(\zeta_{1}\right),\alpha^{\prime \prime \prime }\left(\zeta_{1}\right),\alpha \left(\zeta_{2}\right),\alpha \left(\zeta_{3}\right),\cdots ,\alpha \left(\zeta_{n}\right),\alpha \left(\zeta_{n+1}\right),\alpha^{\prime }\left(\zeta_{n+1}\right),\alpha^{\prime \prime }\left(\zeta_{n+1}\right),\alpha^{\prime \prime \prime }\left(\zeta_{n+1}\right)\;.$$

Following the approach presented in [46], the weighting coefficients of the first four derivatives are defined as

(34) $$A=N_{0}^{\prime }N_{0}^{-1}\;,\;B=\mathrm{AA}\;,\;G=\mathrm{AAA}\;,\;D=\mathrm{AAAA}\;.$$

The discretized version of Equation (20) is then

(35) $$Lw=\Omega^{2}Hw\;,$$

where the matrices L and H are the discretized versions of the differential operators

(36) $$L=16f{\left(\zeta \right)}^{q_{1}+2}\frac{\partial^{4}}{\partial \zeta^{4}}+\epsilon 16\left(2+q_{1}\right)f{\left(\zeta \right)}^{q_{1}+1}\frac{\partial^{3}}{\partial \zeta^{3}}+\epsilon^{2}4\left(q_{1}+1\right)\left(q_{1}+2\right)f{\left(\zeta \right)}^{q_{1}}\frac{\partial^{2}}{\partial \zeta^{2}}\;,$$

and

(37) $$H=-4\eta^{2}f{\left(\zeta \right)}^{q_{2}}\frac{\partial^{2}}{\partial \zeta^{2}}-\eta^{2}\epsilon 4q_{2}f{\left(\zeta \right)}^{q_{2}-1}\frac{\partial }{\partial \zeta }-\eta^{2}\epsilon^{2}q_{2}\left(q_{2}-1\right)f{\left(\zeta \right)}^{q_{2}-2}+f{\left(\zeta \right)}^{q_{2}}\;,$$

and whose entries are

(38) $$L_{i,j}=16f_{i}^{q_{1}+2}D_{i,j}+\epsilon \;16\left(2+q_{1}\right)f_{i}^{q_{1}+1}G_{i,j}+\epsilon^{2}4\left(q_{1}+1\right)\left(q_{1}+2\right)f_{i}^{q_{1}}B_{i,j}\;,$$

and

(39) $$H_{i,j}=-4\eta^{2}f_{i}^{q_{2}}B_{i,j}-\eta^{2}\epsilon \;4q_{2}\;f_{i}^{q_{2}-1}A_{i,j}-\left(\eta^{2}\epsilon^{2}q_{2}\left(q_{2}-1\right)f_{i}^{q_{2}-2}-f_{i}^{q_{2}}\right)\delta_{ij}\;,$$

where $\delta_{ij}$ is the Kronecker operator.

The corresponding boundary conditions are

(40) $$\begin{array}{c} v_{1}=0\;, \end{array}$$

(41) $$\begin{array}{c} v_{1}^{\prime }=0\;, \end{array}$$

(42) $$\begin{array}{c} f_{n+1}^{q_{1}+2}\;v_{n+1}^{\prime \prime }+K_{R}\;v_{n+1}^{\prime }=-\frac{\eta^{2}}{4}f_{n+1}^{q_{2}\;}v_{n+1}\;, \end{array}$$

(43) $$\begin{array}{c} \begin{array}{cc} & \left(\frac{\epsilon }{2}\left(2+q_{1}\right)f_{n+1}^{q_{1}+1}\;v_{n+1}^{\prime \prime }+f_{n+1}^{q_{1}+2}v_{n+1}^{\prime \prime \prime }\right)-K_{T}\;v_{n+1}\\ & =-\eta^{2}\left(\epsilon \;\frac{1}{8}q_{2}\;f_{n+1}^{q_{2}-1}\;v_{n+1}+\frac{1}{4}f_{n+1}^{q_{2}}\;v_{n+1}^{\prime }\right)-\frac{\lambda }{8}\;v_{n+1}\;. \end{array} \end{array}$$

By swapping, in the matrices L and H, the (n+6)th and (n+7)th rows (columns) with the third and fourth rows (columns), Equation (35) can be rearranged as

(44) $$\left(\begin{array}{cc} L_{\mathrm{aa}} & L_{\mathrm{ab}}\\ L_{\mathrm{ba}} & L_{\mathrm{bb}} \end{array}\right)\left(\begin{array}{c} w_{C}\\ w_{F} \end{array}\right)=\Omega^{2}\left(\begin{array}{cc} 0 & H_{\mathrm{ab}}\\ H_{\mathrm{ba}} & H_{\mathrm{bb}} \end{array}\right)\left(\begin{array}{c} w_{C}\\ w_{F} \end{array}\right)\;,$$

where

(45) $$w_{C}=\left(\begin{array}{c} v_{1}\\ v_{1}^{\prime }\\ v_{n+1}^{\prime \prime }\\ v_{n+1}^{\prime \prime \prime } \end{array}\right)\;,\;w_{F}=\left(\begin{array}{c} v_{2}\\ v_{3}\\ \cdots \\ v_{n+1}\\ v_{n+1}^{\prime }\\ v_{1}^{\prime \prime }\\ v_{1}^{\prime \prime \prime } \end{array}\right)\;.$$

The only non-zero elements of $L_{\mathrm{aa}}$ and $L_{\mathrm{ab}}$ are

(46) $$\begin{array}{cc} \; & L_{aa}\left(1,1\right)=L_{aa}\left(2,2\right)=1\;,\\ \; & L_{aa}\left(3,3\right)=f_{n+1}^{q_{1}+2}\;,\\ \; & L_{aa}\left(4,3\right)=\frac{\epsilon }{2}\left(2+q_{1}\right)f_{n+1}^{q_{1}+1}\;,\\ \; & L_{aa}\left(4,4\right)=f_{n+1}^{q_{1}+2}\;,\\ \; & L_{ab}\left(3,n+5\right)=K_{R}\;,\\ \; & L_{ab}\left(4,n+4\right)=-K_{T}\;, \end{array}$$

whereas $L_{\mathrm{ba}}$ and $L_{\mathrm{bb}}$ are

(47) $$L_{\mathrm{ba}}=\left(\begin{array}{cccc} L_{5,1} & L_{5,2} & L_{5,n+6} & L_{5,n+7}\\ \cdots & \cdots & \cdots & \cdots \\ L_{n+4,1} & L_{n+4,2} & L_{n+4,n+6} & L_{n+4,n+7}\\ L_{n+5,1} & L_{n+5,2} & L_{n+5,n+6} & L_{n+5,n+7}\\ L_{3,1} & L_{3,2} & L_{3,n+6} & L_{3,n+7}\\ L_{4,1} & L_{4,2} & L_{4,n+6} & L_{4,n+7} \end{array}\right)\;,$$

(48) $$L_{\mathrm{bb}}=\left(\begin{array}{cccccc} L_{5,5} & \cdots & L_{5,n+4} & L_{5,n+5} & L_{5,3} & L_{5,4}\\ \cdots & \cdots & \cdots & \cdots & \cdots & \cdots \\ L_{n+4,5} & \cdots & L_{n+4,n+4} & L_{n+4,n+5} & L_{n+4,3} & L_{n+4,4}\\ L_{n+5,5} & \cdots & L_{n+5,n+4} & L_{n+5,n+5} & L_{n+5,3} & L_{n+5,4}\\ L_{3,5} & \cdots & L_{3,n+4} & L_{3,n+5} & L_{3,3} & L_{3,4}\\ L_{4,5} & \cdots & L_{4,n+4} & L_{4,n+5} & L_{4,3} & L_{4,4} \end{array}\right)\;.$$

The only non-zero elements of $H_{\mathrm{ab}}$ are given by

(49) $$\begin{array}{cc} \; & H_{ab}\left(3,n+4\right)=-\frac{\eta^{2}}{4}f_{n+1}^{q_{2}}\;,\\ \; & H_{ab}\left(4,n+4\right)=-\left(\eta^{2}\epsilon \frac{1}{8}q_{2}f_{n+1}^{q_{2}-1}+\frac{\lambda }{8}\right)\;,\\ \; & H_{ab}\left(4,n+5\right)=-\frac{\eta^{2}}{4}f_{n+1}^{q_{2}}\;, \end{array}$$

whereas $H_{\mathrm{ba}}$ and $H_{\mathrm{bb}}$ are arranged as

(50) $$H_{\mathrm{ba}}=\left(\begin{array}{cccc} H_{5,1} & H_{5,2} & H_{5,n+4} & H_{5,n+5}\\ \cdots & \cdots & \cdots & \cdots \\ H_{3,1} & H_{3,2} & H_{3,n+4} & H_{3,n+5}\\ H_{4,1} & H_{4,2} & H_{4,n+4} & H_{4,n+5}\\ H_{n+6,1} & H_{n+6,2} & H_{n+6,n+4} & H_{n+6,n+5}\\ H_{n+7,1} & H_{n+7,2} & H_{n+7,n+4} & H_{n+7,n+5} \end{array}\right)\;,$$

(51) $$H_{\mathrm{bb}}=\left(\begin{array}{cccccc} H_{5,5} & \cdots & H_{5,3} & H_{5,4} & H_{5,n+6} & H_{5,n+7}\\ \cdots & \cdots & \cdots & \cdots & \cdots & \cdots \\ H_{3,5} & \cdots & H_{3,3} & H_{3,4} & H_{3,n+6} & H_{3,n+7}\\ H_{4,5} & \cdots & H_{4,3} & H_{4,4} & H_{4,n+6} & H_{4,n+7}\\ H_{n+6,5} & \cdots & H_{n+6,3} & H_{n+6,4} & H_{n+6,n+6} & H_{n+6,n+7}\\ H_{n+7,5} & \cdots & H_{n+7,3} & H_{n+7,4} & H_{n+7,n+6} & H_{n+7,n+7} \end{array}\right)\;.$$

Solving Equation (44), we obtain

(52) $$\begin{array}{c} L_{\mathrm{aa}}w_{C}+L_{\mathrm{ab}}w_{F}=\Omega^{2}H_{\mathrm{ab}}w_{F}\;, \end{array}$$

(53) $$\begin{array}{c} L_{\mathrm{ba}}w_{C}+L_{\mathrm{bb}}w_{F}=\Omega^{2}H_{\mathrm{ba}}w_{C}+\Omega^{2}H_{\mathrm{bb}}w_{F}\;. \end{array}$$

Calculating $w_{C}$ from Equation (52) and substituting it into Equation (53), we get

(54) $$\left(\Omega^{4}H_{\mathrm{ba}}L_{\mathrm{aa}}^{-1}H_{\mathrm{ab}}-\Omega^{2}\left(L_{\mathrm{ba}}L_{\mathrm{aa}}^{-1}H_{\mathrm{ab}}-H_{\mathrm{bb}}+H_{\mathrm{ba}}L_{\mathrm{aa}}^{-1}L_{\mathrm{ab}}\right)+L_{\mathrm{ba}}L_{\mathrm{aa}}^{-1}L_{\mathrm{ab}}-L_{\mathrm{bb}}\right)w_{F}=0\;,$$

from which the eigenvalues $\Omega_{i}$ can be obtained by applying the resolution methods proposed in [47].

The proposed method was tested in [35], where the minimum number of grid points assuring the convergence of the results was assessed and, in particular, it was shown that the first and second frequencies of a cantilever CNT are correctly predicted with n=4 and n=6 using the basis provided in Equation (27).

## 4. Numerical Examples

Some numerical examples are reported in this section to evaluate the effects of parameters η, ϵ, λ, and $K_{R},$ on the resonance frequency of a nonuniform nanobeam. The calculations were performed using in-house DQ software developed in *Mathematica*^®^ language [48] and the results were validated by comparison with those available in the literature. The properties of the considered nanobeam, shown in Table 1, were taken from [36], to which we refer for further details on their derivation.

### 4.1. Effect of the Taper Ratio Coefficient ϵ on Frequency

Here, we analyze the influence of the taper ratio on the natural frequency of nonuniform nanobeams, under the assumptions that nonlocal effects are negligible (η=0) and no lumped mass is present (λ=0). Values of the first nondimensional frequency $\Omega_{1},$ for different values of ϵ are reported in Table 2, with the other parameters relevant for computation provided in the caption. To verify the accuracy and validity of the proposed approach, numerical and exact results are compared, the latter from the solution obtained in [49] using Bessel functions. We can observe that the DQM results are very accurate approximations of exact ones, with very small, or even vanishing, relative errors computed as [50]

(55) $$\mathrm{err}=\left|\frac{\Omega_{1,\mathrm{DQM}}-\Omega_{1,\mathrm{exact}}}{\Omega_{1,\mathrm{exact}}}\right|\;.$$

### 4.2. Effect of a Lumped Mass Applied to the Tip

A lumped mass placed at the tip of nonuniform nanobeams is considered in this section, and its influence on the natural frequency is analyzed. Assuming that λ=0.5 holds, the values of the first nondimensional frequency $\Omega_{1},$ for different values of ϵ are reported in Table 3, with the other parameters relevant for computation provided in the caption. As in the previously reported examples, the numerical and exact results are compared, and we found an excellent agreement. Note that at a precision of four digits, the numerical and exact results coincide, but for ϵ=1, the relative errors (Equation (55)) are however very low.

### 4.3. Effect of the Nonlocal Parameter η on Frequency

The basic principle of mass sensors relies on quantifying the difference between the fundamental frequency of the CNT or CNC with and without the attached mass. The relative frequency shift, which is given by

(56) $$\Delta f=\frac{\omega_{0}-\omega_{\mathrm{nl}}}{2\pi }=f_{0}-f_{\mathrm{nl}}\;,$$

where $f_{\mathrm{nl}}$ and $f_{0}$ are the natural frequencies of the nanobeam with and without added mass and nonlocal effect, respectively, can be exploited to determine the value of the attached mass [51]. The effect of the nonlocal parameter on frequency shift is investigated.

In Table 4, the resonant frequency shift values are reported for three different values of ϵ, namely −0.5, 0, and 0.5, with the other parameters relevant for computation provided in the caption. The frequency shift decreases for increasing ϵ and η.

**Figure 2:**
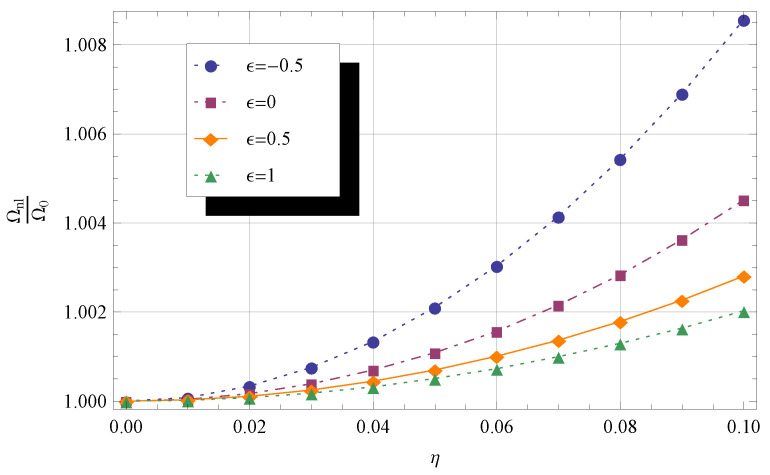
Frequency ratio $\Omega_{\mathrm{nl}}/\Omega_{0}$ for different values of η and ϵ. The other parameters are $q_{1}=1,$ $q_{2}=1,$ λ=0, $K_{R}=0,$ and $K_{T}=0$.

**Figure 3:**
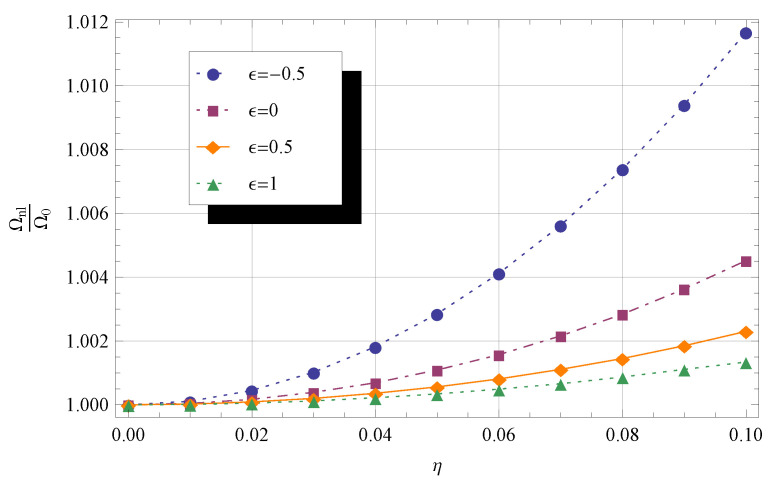
Frequency ratio $\Omega_{\mathrm{nl}}/\Omega_{0}$ for different values of η and ϵ. The other parameters are $q_{1}=2,$ $q_{2}=2,$ λ=0, $K_{R}=0,$ and $K_{T}=0$.

In Figure 2 and Figure 3, the frequency ratio $\Omega_{\mathrm{nl}}/\Omega_{0}$ is plotted against the nonlocal parameter η, with 0⩽η⩽0.1, λ=0, and ϵ taking four values, namely −0.5, 0, 0.5, and 1. In Figure 2, the cross-sectional area (Equation (13)) and second moment of area (Equation (14)) profiles are governed by $q_{2}=1$ and $q_{1}=1,$ respectively, whereas in Figure 3, $q_{2}=2$ and $q_{1}=2$ are set. Note that the values of $\Omega_{\mathrm{nl}}/\Omega_{0}$ are higher in the latter case than in the former.

### 4.4. Effect of the Dimensionless Rotational Stiffness $K_{R}$ on Frequency

The effect of the dimensionless rotational stiffness $K_{R}$ on frequency is considered here. The results in Table 5, with the parameters relevant for computation reported in the caption, show that the first three dimensionless frequencies $\Omega_{\mathrm{nl}}$ increase with $K_{R}$, then remain constant for values of $K_{R}$ greater than $10^{3},$ corresponding to a fixed rotational constraint.

### 4.5. Effect of the Dimensionless Parameter λ and Taper Ratio ϵ on Frequency Shift

**Figure 4:**
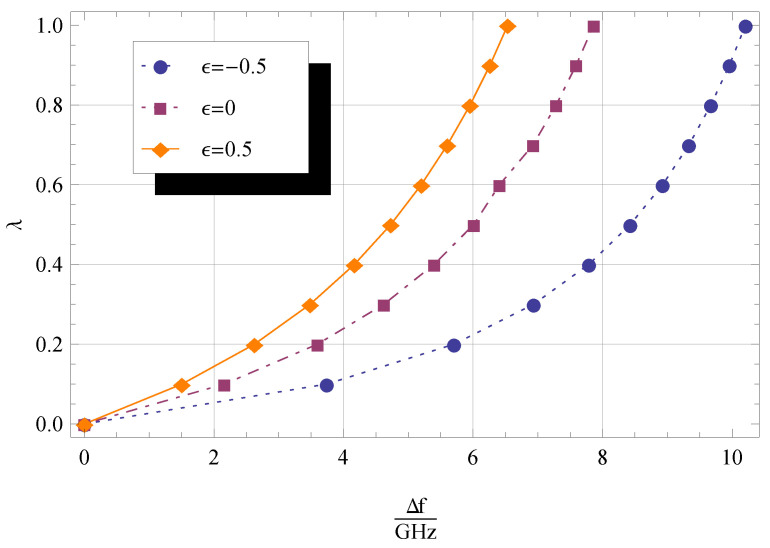
Dimensionless frequency shift Δf/GHz for 0≤λ≤1, and three different values of ϵ. The other parameters are $q_{1}=1,$ $q_{2}=1,$ η=0.1, $K_{R}=0,$ and $K_{T}=0$.

**Figure 5:**
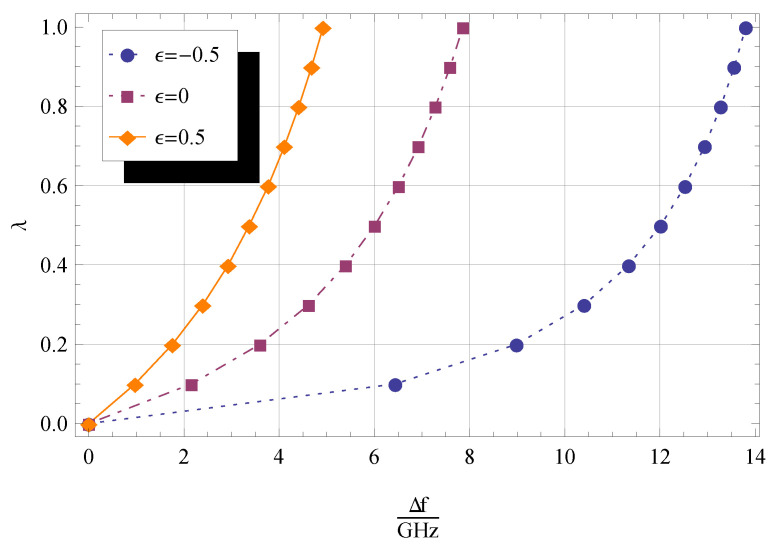
Dimensionless frequency shift Δf/GHz for 0≤λ≤1, and three different values of ϵ. The other parameters are $q_{1}=2,$ $q_{2}=2,$ η=0.1, $K_{R}=0,$ and $K_{T}=0$.

The influence of λ and η on frequency shift is graphically shown in Figure 4 and Figure 5, from which we can see that Δf increases for decreasing ϵ and increasing λ. Moreover, it can be argued that

i for ϵ=0.5, the influence of the added mass on values of Δf is more pronounced for $q_{1}=q_{2}=2$ (Figure 4) than for $q_{1}=q_{2}=1$ (Figure 5);
ii for ϵ=−0.5, values of Δf evaluated for $q_{1}=q_{2}=2$ (Figure 4) are greater than those for $q_{1}=q_{2}=1$ (Figure 5);
iii Δf tends asymptotically to a constant value as λ increases.

## 5. Conclusions

In this study, the nonlocal free vibration analysis of nanobeams, modeling CNTs or CNCs at the continuum level, was considered. The nanobeams are clamped at one end and elastically restrained at the other, where a lumped mass is also applied. The equation of motion and its boundary conditions were derived according to the non-local Euler-Bernoulli beam theory and then solved using the differential quadrature method (DQM). The accuracy of the proposed method was investigated by comparing numerical and exact results. The effects of several parameters, namely taper ratio, nonlocal parameter, lumped mass, and elastic boundary conditions, on free frequencies were discussed. Through the obtained results, the following observations were obtained:

i for a fixed value of λ, the frequency shift decreases as the nonlocal parameter η and the taper ratio ϵ increase;
ii if the rotational stiffness $K_{R}$ increases, the first three dimensionless frequencies $\Omega_{\mathrm{nl}}$ increase and, for $K_{R}>10^{3}$, settle at a fixed value;
iii for fixed values of η and ϵ and increasing λ, the frequency shift increases toward an asymptotic value.

The results also show that the DQM provides an excellent approximation of the exact solution. The accuracy of the results confirm that the proposed algorithm provides a simple and powerful tool in dealing with the free vibration analysis of nanobeams.

## Figures and Tables

**Table 1:** Geometrical and material properties adopted in the numerical experiments.

Properties	Symbol	Value	Unit
Length	L	$2.200\times 10^{-8}$	m
Cross-sectional area	A${}_{0}$	1.70903 $\times \;10^{-18}$	m${}^{2}$
Second moment of area	$I_{0}$	5.71584 $\times \;10^{-37}$	m${}^{4}$
Mass density	ρ	$2.240\times 10^{3}$	kg m${}^{-3}$
Young’s modulus	E	1.000	TPa

**Table 2:** Comparison of the first dimensionless frequency $\Omega_{1}$ from the exact solution [49] and DQM for different ϵ. The other parameters are $q_{1}=2,$ $q_{2}=2,$ η=0, λ=0, $K_{R}=0,$ and $K_{T}=0$.

ϵ	$\Omega_{1,\mathrm{exact}}$ [49]	$\Omega_{1,\mathrm{DQM}}$	err
1.0	1.6113	1.6114	$6.2062\times 10^{-5}$
0.9	1.6301	1.6302	$6.1346\times 10^{-5}$
0.8	1.6500	1.6501	$6.0606\times 10^{-5}$
0.7	1.6712	1.6714	$11.967\times 10^{-5}$
0.6	1.6940	1.6940	0.
0.5	1.7183	1.7184	$5.8197\times 10^{-5}$
0.4	1.7445	1.7447	$11.465\times 10^{-5}$
0.3	1.7730	1.7730	0.
0.2	1.8039	1.8039	0.

**Table 3:** Comparison of the first dimensionless frequency $\Omega_{1}$ from the exact solution [49] and DQM for different ϵ. The other parameters are $q_{1}=2,$ $q_{2}=2,$ η=0, λ=0.5, $K_{R}=0,$ and $K_{T}=0$.

ϵ	$\Omega_{1,\mathrm{exact}}$ [49]	$\Omega_{1,\mathrm{DQM}}$	err
1.0	1.4421	1.4422	$6.9343\times 10^{-5}$
0.9	1.4459	1.4459	0.
0.8	1.4489	1.4489	0.
0.7	1.4511	1.4511	0.
0.6	1.4522	1.4522	0.
0.5	1.4520	1.4520	0.
0.4	1.4503	1.4503	0.
0.3	1.4466	1.4466	0.
0.2	1.4407	1.4407	0.

**Table 4:** Frequency shift Δf for different values of η and ϵ. The other parameters are $q_{1}=1,$ $q_{2}=1,$ λ=0.5, $K_{R}=0,$ and $K_{T}=0$.

η	ϵ=−0.5	ϵ=0	ϵ=0.5
0	8.4456	6.0259	4.7428
0.02	8.4449	6.0254	4.7423
0.04	8.4428	6.0236	4.7407
0.06	8.4393	6.0206	4.7382
0.08	8.4344	6.0163	4.7346
0.10	8.4281	6.0109	4.7300
0.12	8.4203	6.0042	4.7244
0.14	8.4110	5.9963	4.7177
0.16	8.4003	5.9871	4.7099
0.18	8.3880	5.9766	4.7011
0.20	8.3740	5.9648	4.6911

**Table 5:** The first three dimensionless frequency $\Omega_{\mathrm{nl}},$ for different values of $K_{R}$ and ϵ. The other parameters are $q_{1}=1,$ $q_{2}=1,$ η=0.1, λ=0.5, and $K_{T}=0$.

$K_{R}$	ϵ=−0.5	ϵ=0	ϵ=0.5
	1.7227	2.0201	2.1908
0	12.2826	15.9842	19.18635
	31.6509	43.2472	53.5648
	1.9472	2.1502	2.2813
0.1	12.9167	16.2385	19.3274
	32.6881	43.4459	53.6609
	2.4073	2.7696	2.8486
1	14.9778	17.9400	20.4497
	35.1213	44.9686	54.4768
	2.5905	3.4929	4.0616
10	16.2189	21.7424	25.2449
	37.4361	50.0820	59.3719
	2.6145	3.6547	4.5134
$10^{2}$	16.4043	23.0538	28.7191
	37.8203	52.5596	65.0277
	2.6171	3.6729	4.5717
$10^{3}$	16.4237	23.2150	29.2433
	37.8610	52.8892	66.1246

## Data Availability

The data presented in this study are available on request from the corresponding author.
